# Supplementary figures and images for: Low and high beta rhythms have different motor cortical sources and distinct roles in movement control and spatiotemporal attention
Source: PLoS Biol. 2024 Jun 25;22(6):e3002670. doi: 10.1371/journal.pbio.3002670 (PMC11198906; doi:10.1371/journal.pbio.3002670)

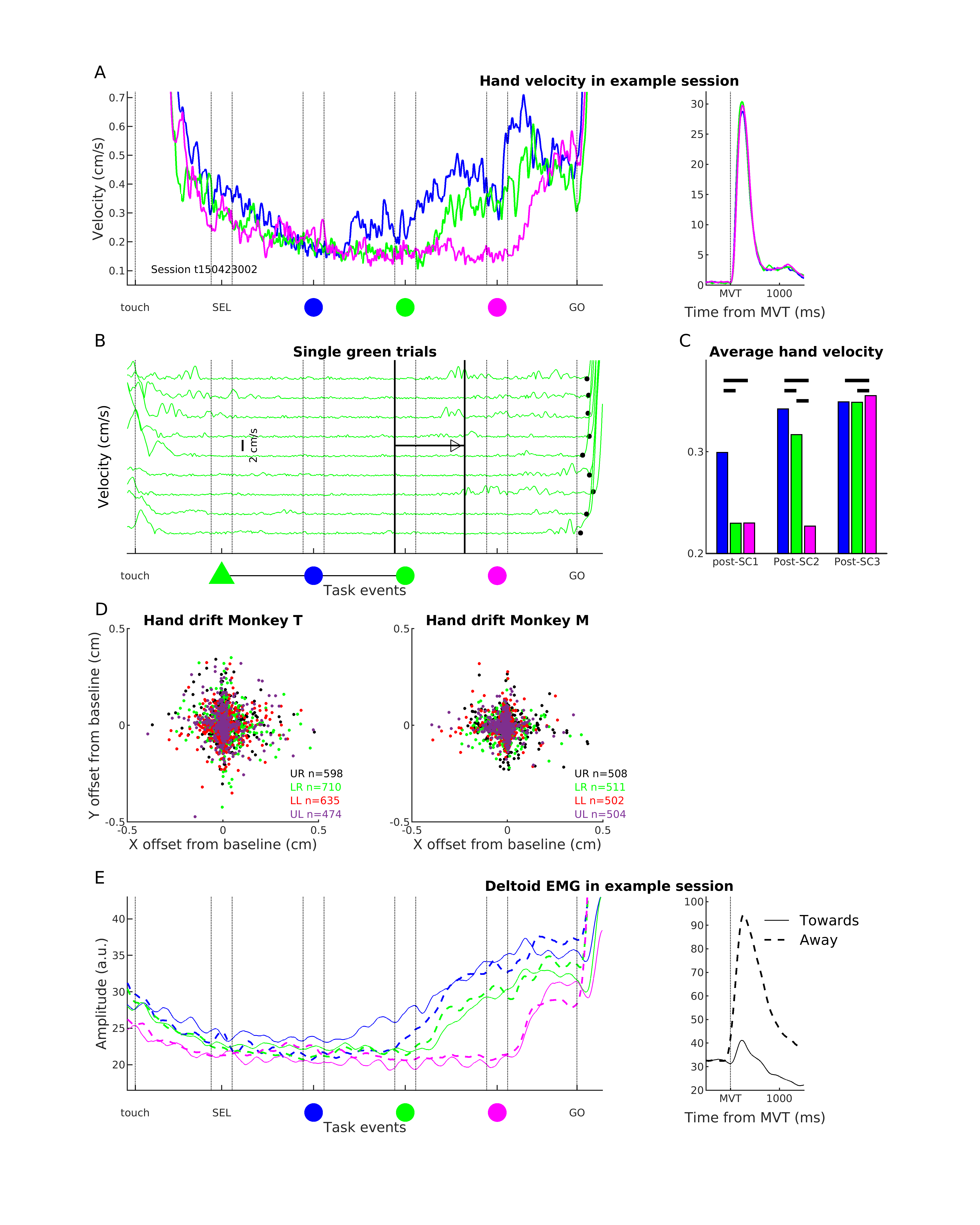

Supplement: S1 Fig — (A) Average hand velocity in 1 example session in monkey T, split for the 3 color conditions. On the left, zoomed in to the micro-movements performed during the trial between central touch and GO. To the right with velocity scale adjusted to the final center-out reaching, aligned to movement onset. (B) Hand velocity in a randomly selected subset of correct green trials in the same session as in A. The 2 solid black vertical lines connected with a horizontal arrow reflect the epoch used to estimate X and Y offset (drift) of micromovements in the post-cue epoch (in D). The velocity scale is indicated inside the plot. (C) Average hand velocity in the 1-s delay after each SC, split for color condition, averaged across all trials for all behavioral sessions for the 2 monkeys combined. Horizontal black lines on top of the bar plots denote significant differences in single-trial hand velocity. (D) Hand cursor displacement (drift) caused by micro-movements across all green trials in each monkey, split according to the target direction. Each dot reflects 1 trial, and the position reflects the relative X and Y offset 1 s after the onset of SC2 (second vertical solid black line in B), compared to the position at SC2 onset (first vertical solid black line in B). UR, upper right; LR, lower right; LL, lower left; UL, upper left. The total number of trials is indicated (n). (E) Average deltoid EMG amplitude, recorded in the same behavioral session as shown in A and B, on the left for the period between touch and GO (split for the 3 color conditions) and on the right aligned to movement onset (averaged for the 3 color conditions). Towards the body (LL) in solid lines and away from the body (UL) in dotted lines. The raw EMG signal (30 kHz) was first rectified, and then low-pass filtered at 250 Hz and downsampled to 1 kHz. A Gaussian filter (length 150 ms, width 100 ms) was used to smooth single trials before plotting the trial-averaged EMG. Source data are available in S2 Data. (TIF [file pbio.3002670.s001.tif]

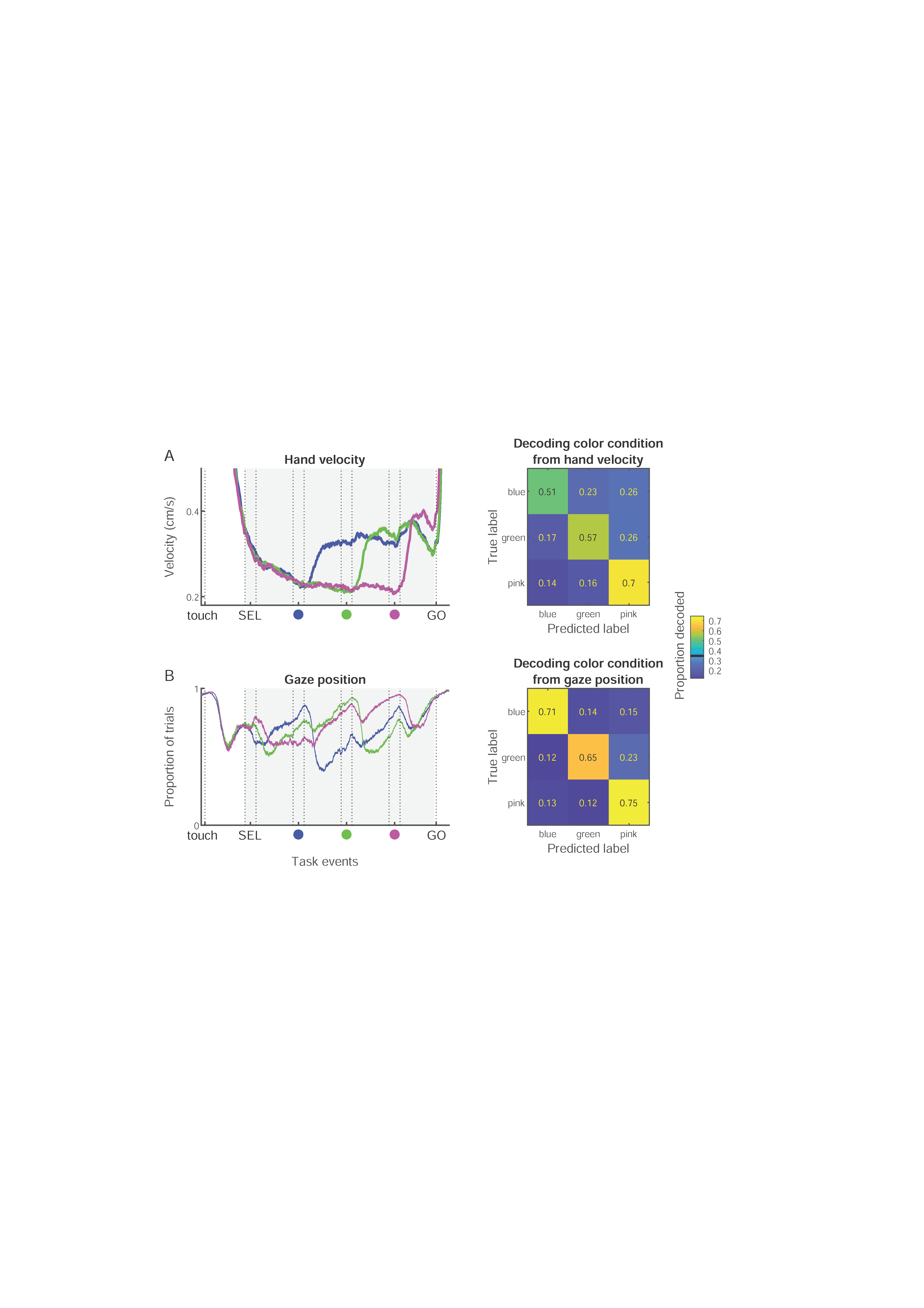

Supplement: S2 Fig — (A) Left. Average hand velocity across all trials in all behavioral sessions. Data from both monkeys are combined (n = 11,587). Gray rectangle represents the epoch considered for the following decoding analysis. Right. Decoding performance of SEL (color condition) category in correct trials using the temporal profile of hand velocity. Performance is presented as proportions of the total number of trials of each category in the test set (totaling 1 for each row). The diagonal represents the true positive accuracy, and the off-diagonal values correspond to the proportions of trials of each category incorrectly assigned to another category. The estimated chance level (0.35) is marked on the color scale bar. (B) Left. Gaze position for both monkeys combined across all trials in all behavioral sessions with eye movement recordings (n = 8,630). Each curve represents the proportion of trials in which the gaze position was inside the working area (number of trials In/number of trials In + Out) along the trial. Eyeblinks were considered as missing data. Right. Decoding performance of SEL (color condition) category in correct trials using the temporal profile of gaze position. Other parameters are the same as in A right. Source data are available in S2 Data. (TIF) [file pbio.3002670.s002.tif]

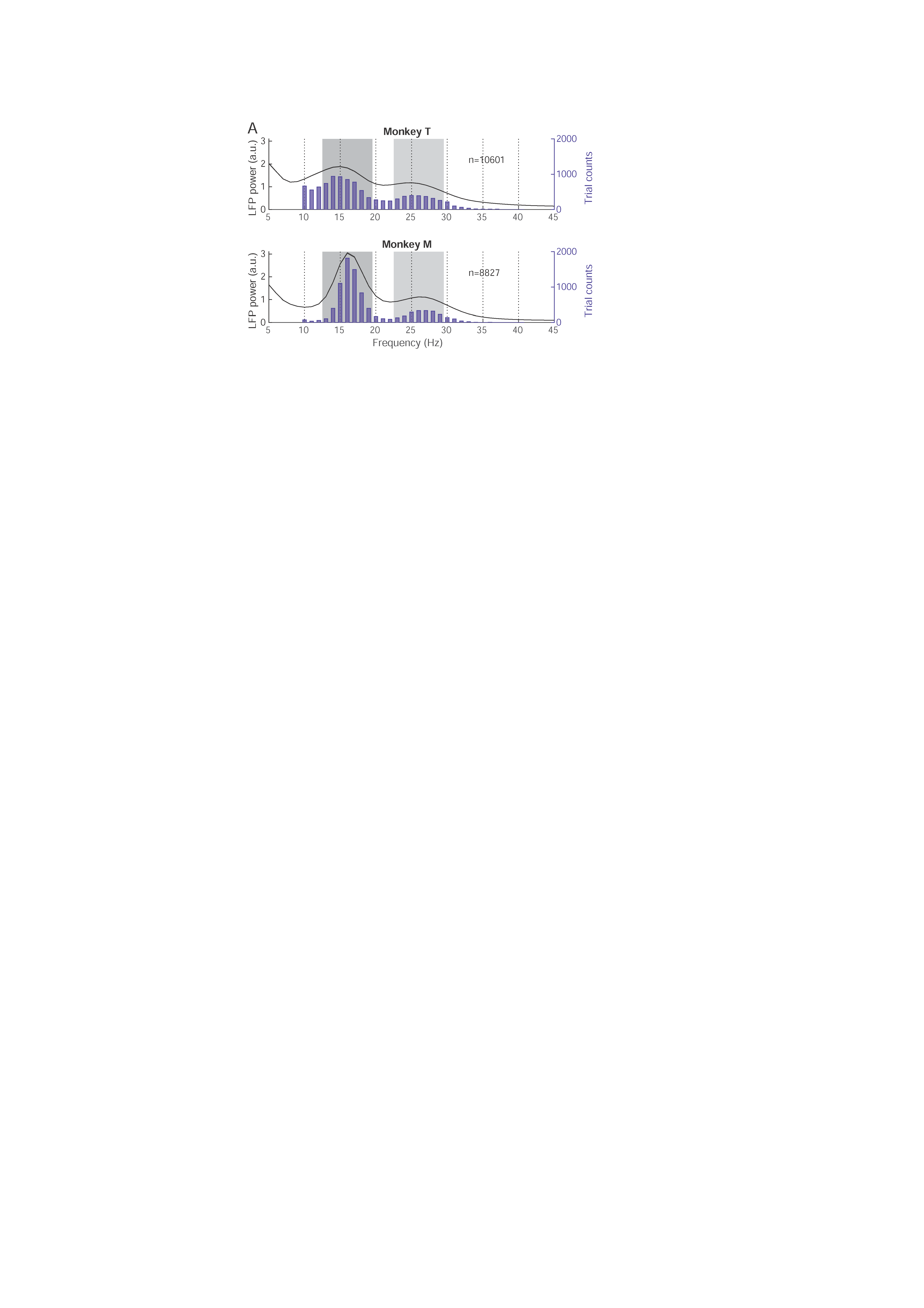

Supplement: S3 Fig — (A) Average normalized power in the pre-SC1 period across all trials for all sites in each monkey, for the full LFP signal, including aperiodic and periodic components. The curves reflect the mean power ±SEM across LFP sites. Overlain are distributions of single-trial peak frequency (frequency with maximal power) between 10 and 40 Hz in the same task period for the full LFP signal. Source data are available in S2 Data. (TIF) [file pbio.3002670.s003.tif]

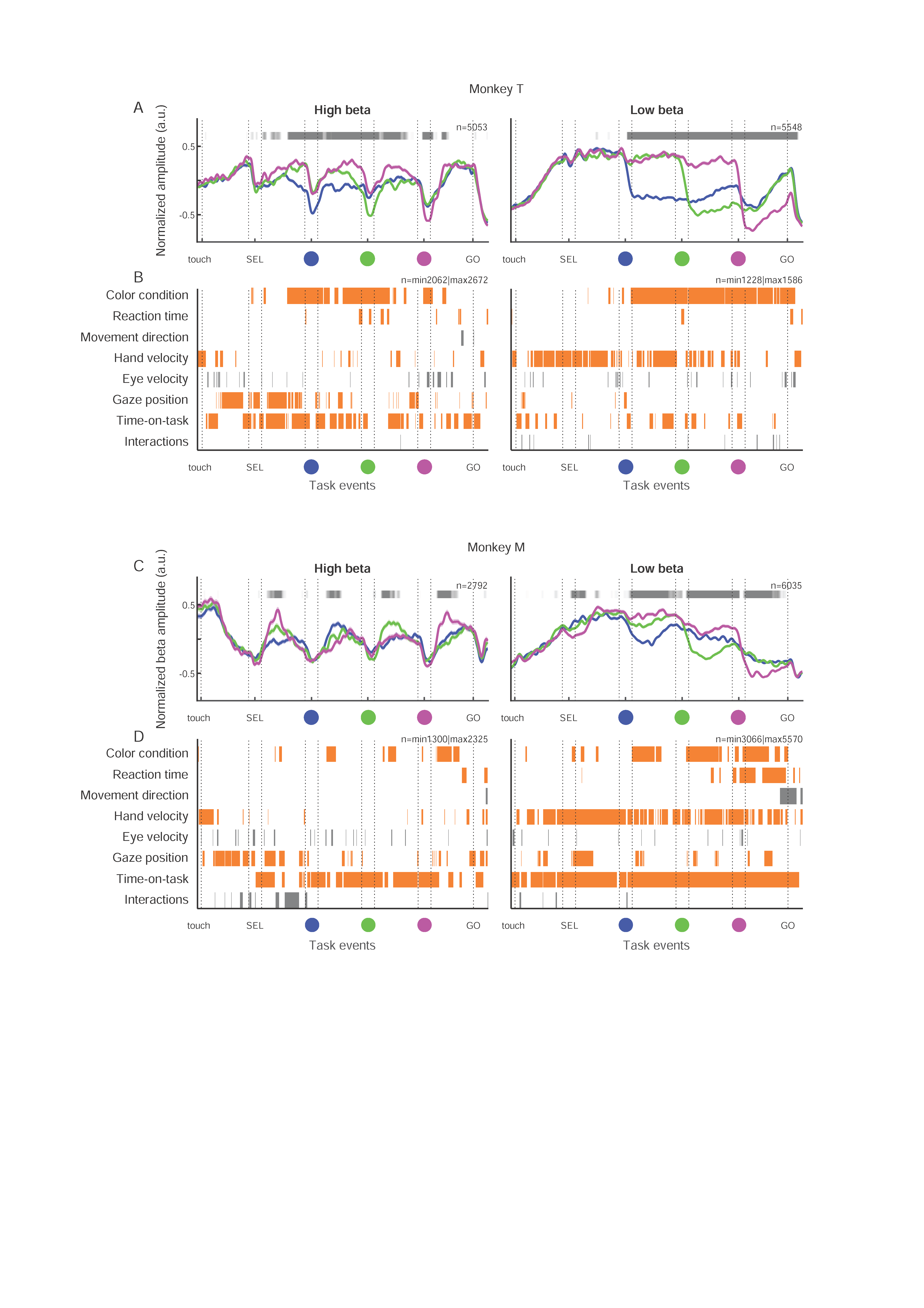

Supplement: S4 Fig — Related to Figs 4 and 6. (A, C) Same as Fig 4A, separated for Monkey T (A) and Monkey M (C). (B, D) Same as Fig 6B, separated for Monkey T (C) and Monkey M (D). (A, C) Representation of the trial-averaged temporal profile of normalized high (left; 21–29 Hz) and low (right; 12–20 Hz) beta amplitude (+/− SEM), separated by the 3 color conditions. The horizontal gray line above each plot graph represents the time-resolved modulation in beta amplitude by the color condition along the task. The significativity is represented as in Fig 4A. (B, D) Time-resolved representation of the presence of each regressor in the winning model after the application of a Bayesian index criterion (BIC) for the comparison of all possible models and their 2-by-2 interactions, for the high beta (left) and the low beta (right). Each row represents a regressor, the last row represents all possible interactions. Regressors selected for ulterior analysis in orange and discarded regressors in gray. Source data are available in S2 Data. (TIF) [file pbio.3002670.s004.tif]

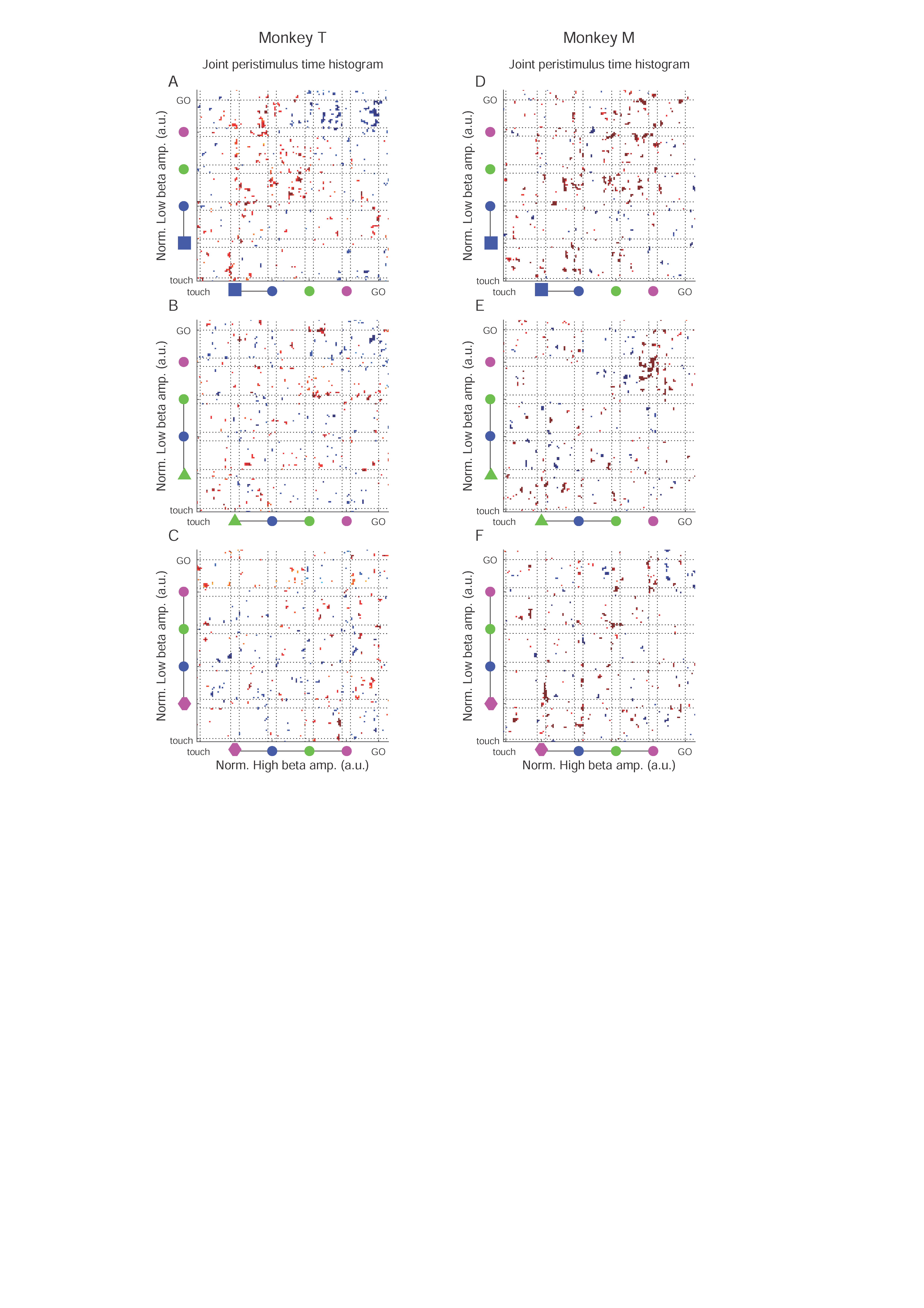

Supplement: S5 Fig — Related to Fig 4. Equivalent of a joint peristimulus time histogram (jpsth) applied to the high and low beta amplitude. The analysis was performed separately for both monkeys (A–C: Monkey T; D–F: Monkey M) and the 3 colors (A, D: blue; B, E: green; C,F: pink). Each point of the matrix represents the corrected trial-by-trial cross product of the 2 variables on 50 ms bins. Each colored matrix point was inferior (cold color) or superior (warm color) to all 100 values from shuffled matrices (equivalent p-value of 0.01). The vertical and horizontal lines represent the appearance and disappearance of the valid SC for the 3 conditions. Vertical color bars on the right of each jpsth represent the significativity of the correlation between low beta and hand velocity, for each color condition. The significativity is represented as a color-scale gradient (brightest color for p = 0.01 and darkest color for p< = 1e-08; white means nonsignificant). Source data are available in S2 Data. (TIF) [file pbio.3002670.s005.tif]

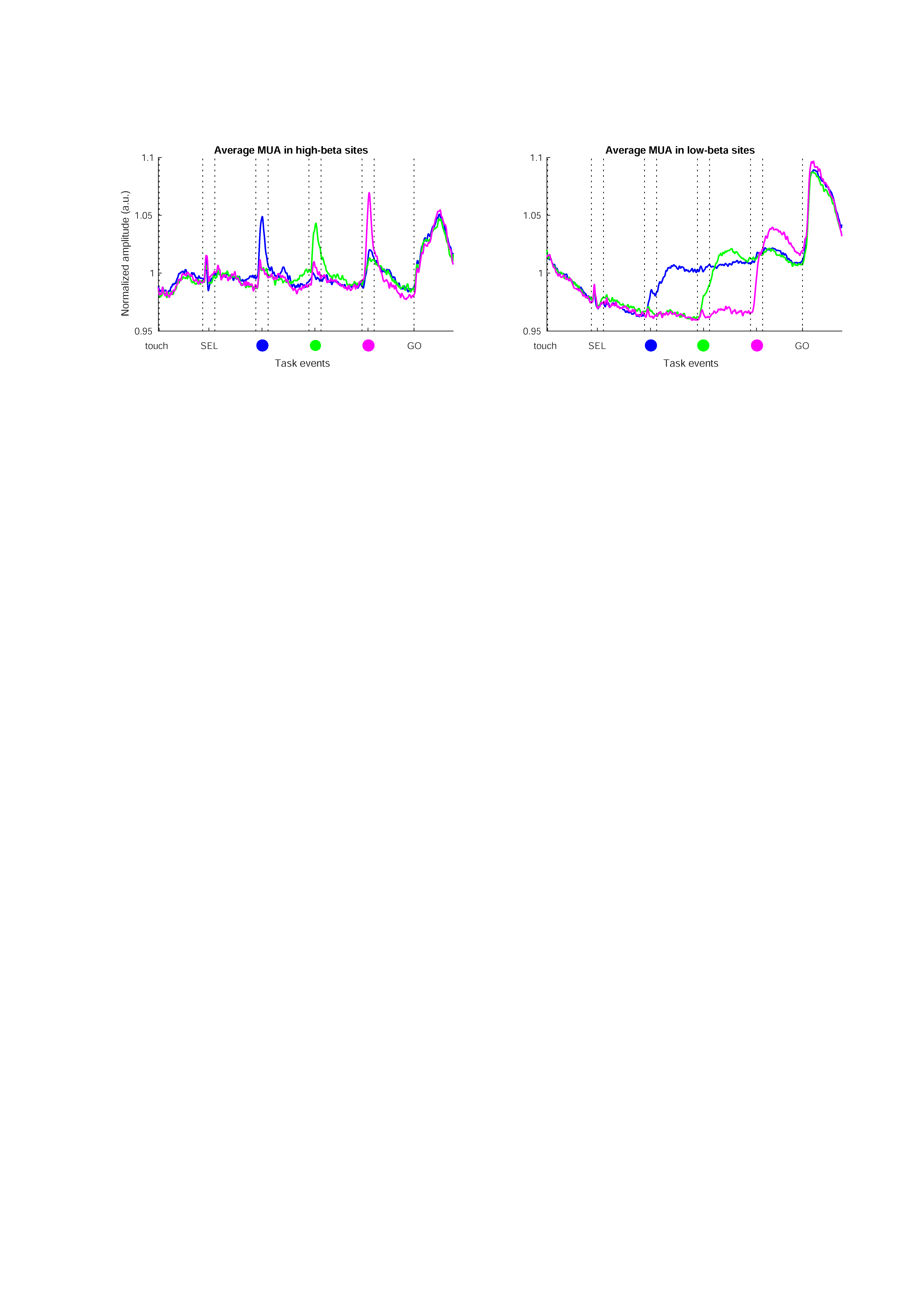

Supplement: S6 Fig — Related to Fig 4. Average MUA amplitude including all trials of all recording sites, for high beta band (left) and low band (right) dominant sites, separately for the 3 color conditions. The MUA was generated following the method of Stark and Abeles (2007) [79]. The raw signal was first bandpass filtered (300–6,000 Hz) and clipped beyond +/− 2 standard deviations. Then, the signal was squared, smoothed with a low-pass filter (250 Hz) and downsampled from 30 to 1 kHz, before the square-root was taken to arrive at the final MUA signal. The MUA from each site was cut in trials, and normalized by dividing by the mean amplitude across all trials and trial-times. Finally, the single-trial MUA from all sites with the same LFP beta band dominance were combined. The plots show trial-averaged MUA after first smoothing individual trials with a Gaussian filter (length 30 ms, width 15 ms). Source data are available in S2 Data. (TIF) [file pbio.3002670.s006.tif]

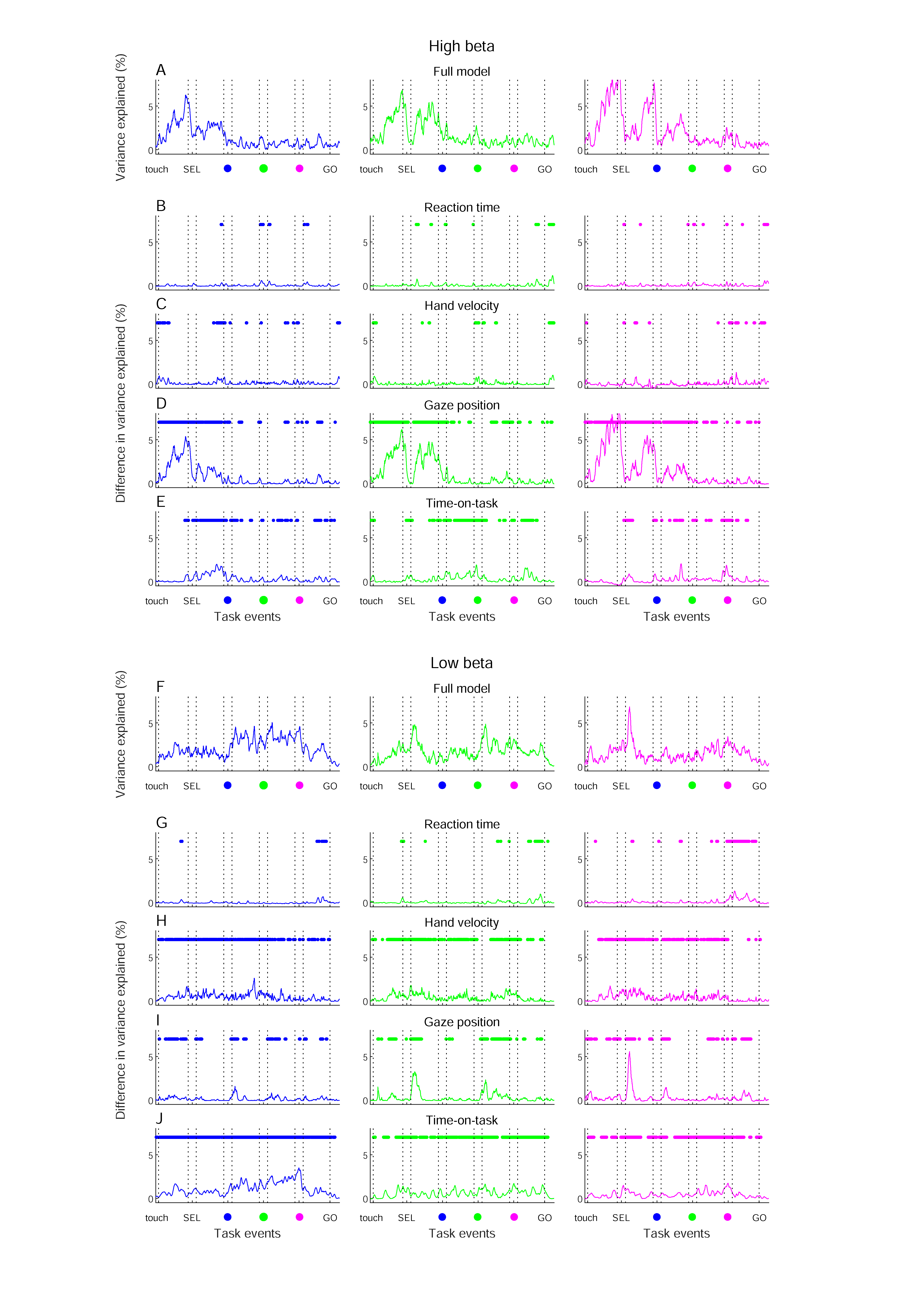

Supplement: S7 Fig — Related to Fig 6. (A) Total percentage of variance explained by the 4 selected regressors, RT, hand Velocity, gaze position, and time-on-task, along the task, in the 3 different color conditions for both bands. (B–E) Percentage of variance explained by the full model minus the full model in which the values of one regressor were scrambled across trials. For each bin, the values were scrambled 100 times and the average of the 100 scrambles was subtracted to values obtained with the full model. The dots above each graph represent an equivalent p-value of 0.01. For a temporal bin, if the value of variance explained obtained with the full model was superior to all the 100 values obtained with the regressor of interest scrambled, the effect of the regressor in that bin was considered significant and marked with a dot. From B to E, the values were scrambled respectively for the RT, hand velocity, gaze position, and time-on-task. Source data are available in S2 Data. (TIF) [file pbio.3002670.s007.tif]

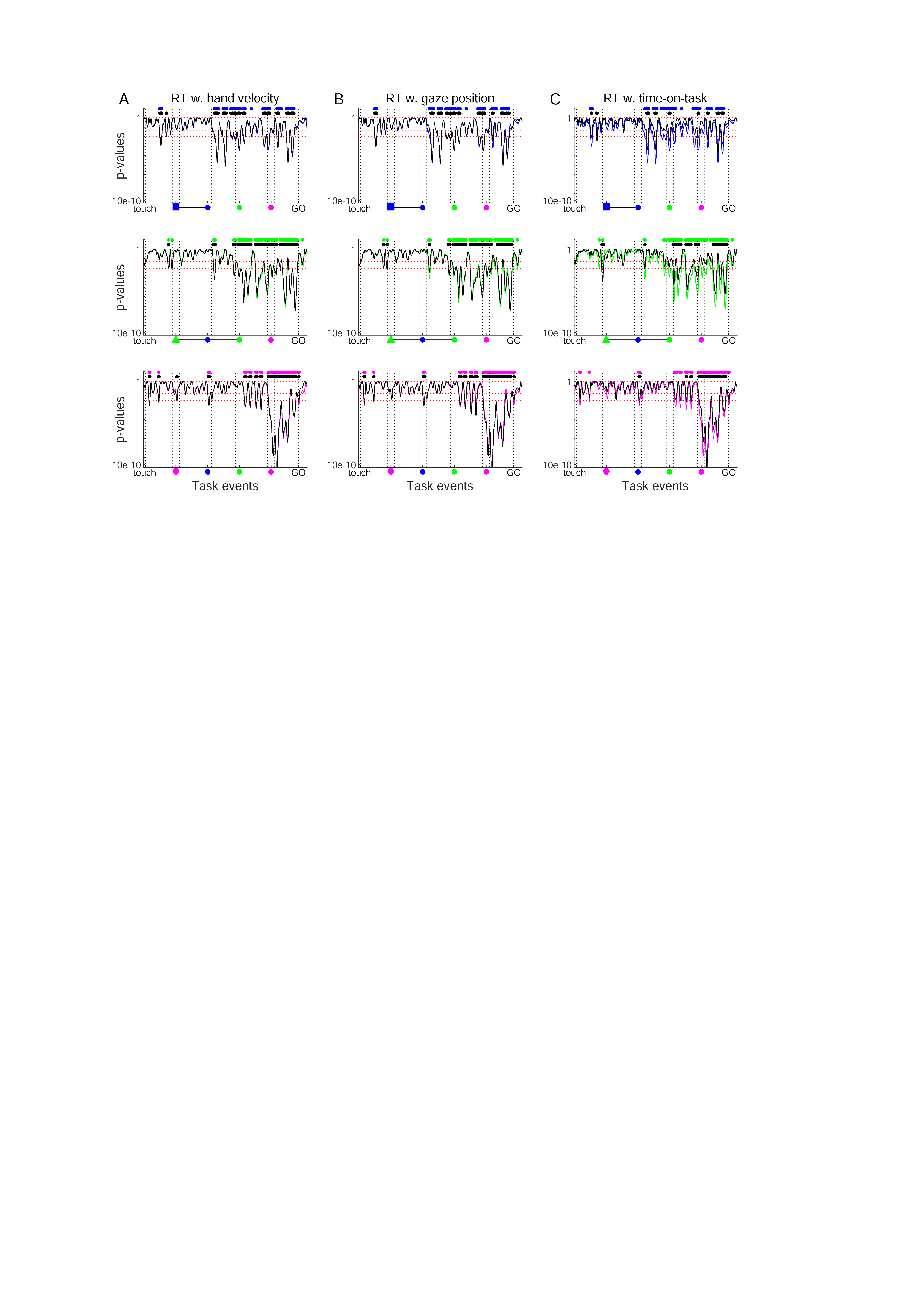

Supplement: S8 Fig — Related to Fig 7. Comparison of the significativity of RT as the unique regressor of a linear model vs. paired with each of the other regressors, separated by color conditions. In each plot, the p-value is displayed on a logarithmic scale, in color when RT was the unique regressor considered and in black when paired with a second regressor. The horizontal dotted lines on the top of each plot represent the significance of each case (p < 0.01). Red horizontal dotted lines are plotted for p-values equal to 1, 0.05 and 0.01. (A) RT paired with hand velocity as a second regressor. (B) RT paired with the gaze position as second regressor. (C) RT paired with time-on-task as second regressor. Source data are available in S2 Data. (TIF) [file pbio.3002670.s008.tif]

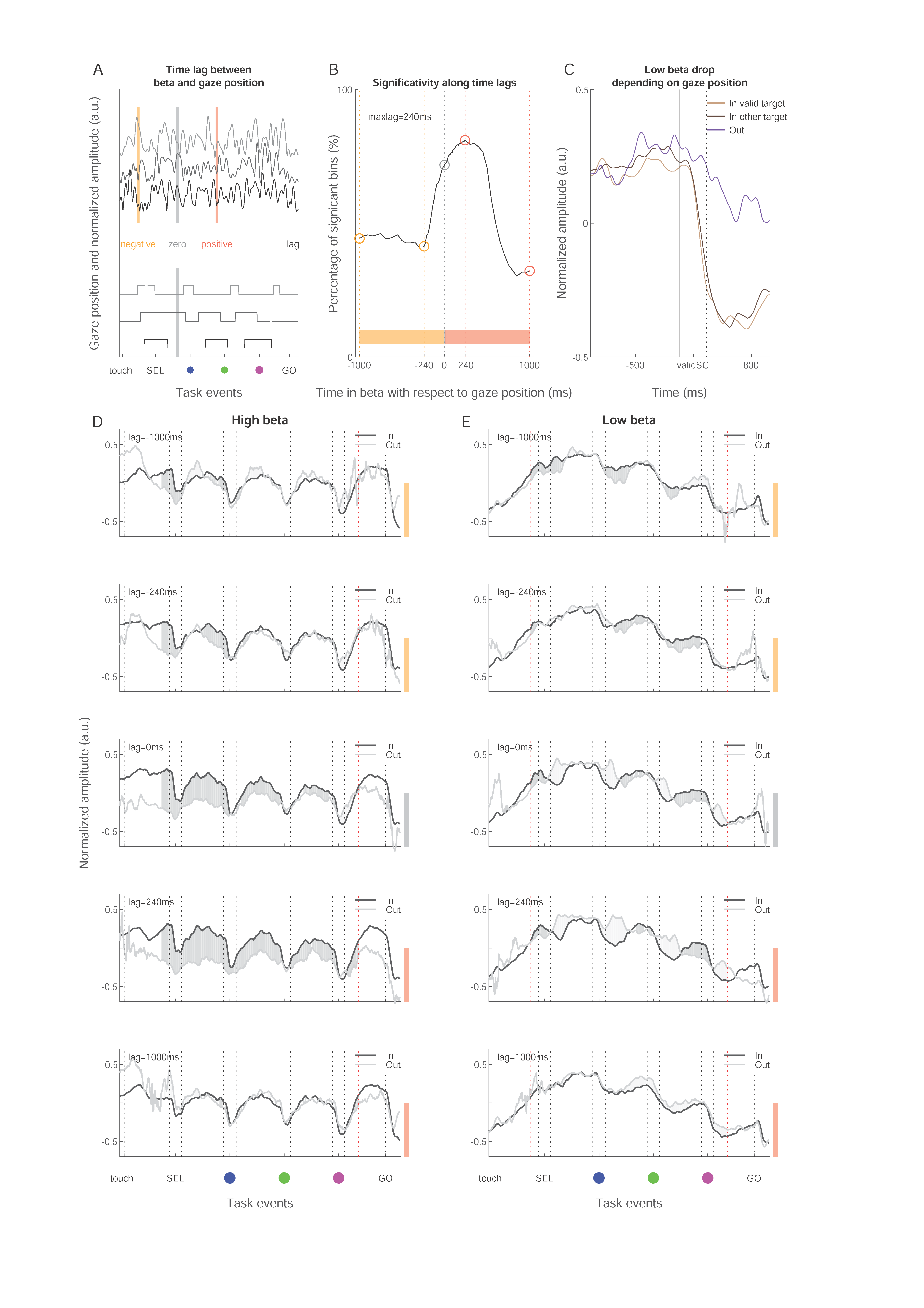

Supplement: S9 Fig — Related to Fig 8. (A) Representation of the negative, zero, and positive lag meaning in the correspondence between LFP (top) and gaze position (bottom). Three trials are represented for beta and gaze position. A negative lag means relating the gaze position with LFP in the past (before), beta was consequently leading gaze. Zero lag means relating LFP and gaze position from the same temporal bin. A positive lag means relating gaze position to the LFP in the future (later), gaze was consequently leading beta. (B) Proportion of bins in which beta amplitude (both bands combined) modulated significantly with gaze position, for different temporal lags. Beta was leading gaze for negative values (i.e., gaze at time t0 and LFP at time t0—lag, represented in orange). Correlation at zero lag is represented in gray. Gaze was leading beta for positive values (i.e., gaze at time t0 and LFP at time t0 + lag, represented in red). (C) Low beta band split into groups of trials based on the position of the gaze at 200 ms after the onset of the valid SC. Brown curves represent the trials in which the monkeys were looking inside the working area (either on the target or elsewhere). Purple represents the trials in which the monkeys were looking outside the working area. (D) High beta amplitude split into groups based on the monkey’s position of the gaze at different time lags (from top to bottom; −1,000 ms, −240 ms, 0 ms, 240 ms, 1,000), all color conditions combined. Red dashed vertical lines represent the values from and up to which significant bins have been counted in B. We excluded the first and last 1,000 ms of the period spanning from −1,200 ms to 5,600 ms from SEL. (E) Same representation for the low beta band. Source data are available in S2 Data. (TIF) [file pbio.3002670.s009.tif]

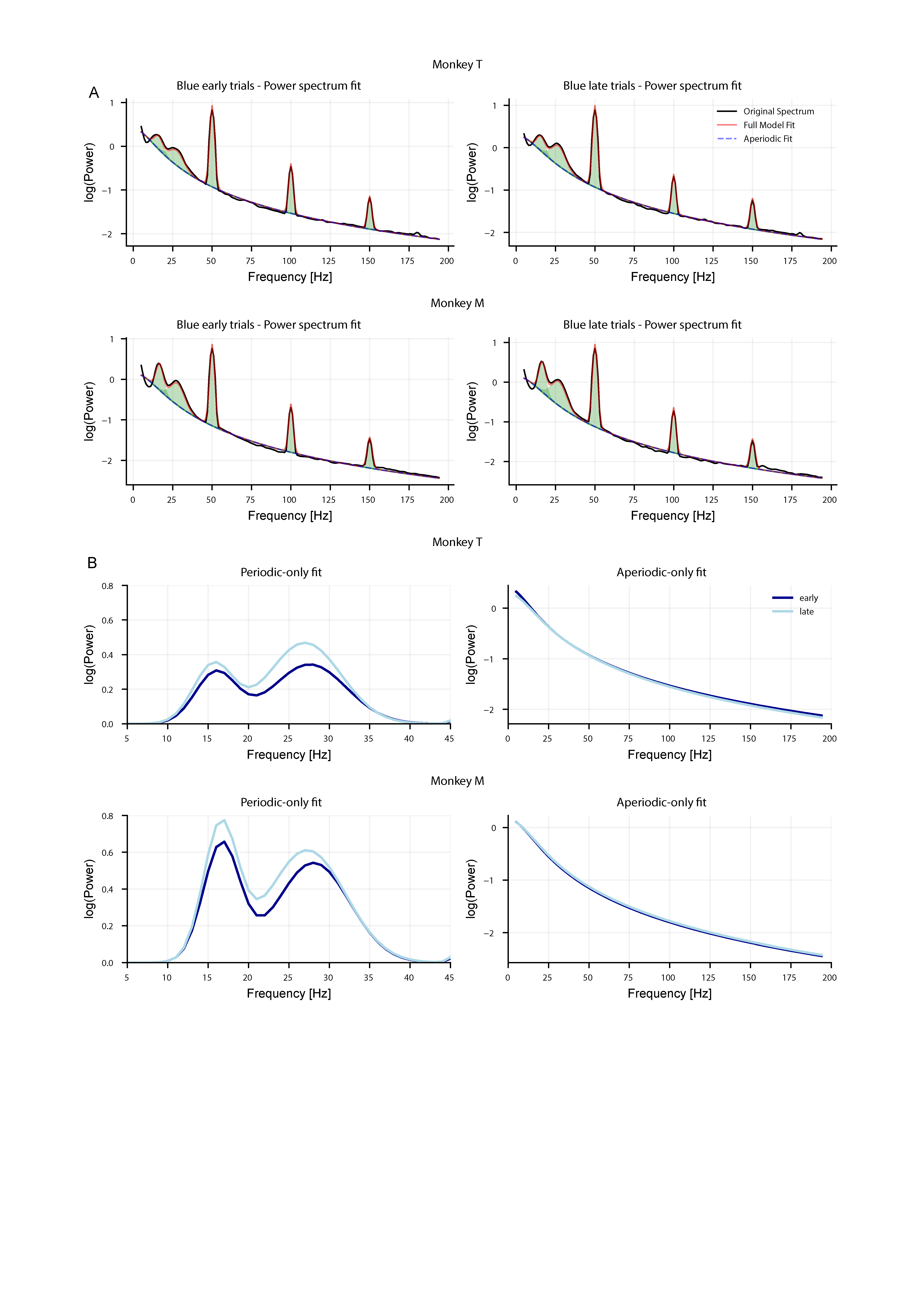

Supplement: S10 Fig — Related to Fig 9. (A) Spectral parametrization using the FOOOF method [73] for the pre-SC1 period of blue trials. The analysis was done separately for the first third of trials for each session (early; left) and the last third of trials for each session late; right), for each monkey separately. The black line corresponds to the original data and the red line to the model fit. The algorithm identifies the aperiodic signal (blue dashed line) and the spectral peaks and their peak frequency (green). A frequency range of 5–194 Hz was used for fitting the data, using the “knee” mode. (B) Spectrum decomposition in periodic (left) and aperiodic (right) signal components, in early and late blue trials in the sessions, for each monkey. The frequency axis was cut at 45 Hz for the periodic signal to focus on the lower frequencies including the beta bands. Source data are available in S2 Data. (TIF) [file pbio.3002670.s010.tif]
