# Supplementary material for: Low and high beta rhythms have different motor cortical sources and distinct roles in movement control and spatiotemporal attention
Source: PLoS Biol. 2024 Jun 25;22(6):e3002670. doi: 10.1371/journal.pbio.3002670 (PMC11198906; doi:10.1371/journal.pbio.3002670)
Supplement: S1 Table — Related to Fig 1. Summary of all errors, number of correct trials included for behavioral analyses, percent of distractor errors and RTs (+/− SD) for each color condition and movement direction for each animal. UR, upper right; LR, lower right; LL, lower left; UL, upper left. (DOCX) [file pbio.3002670.s011.docx]

| **Overview of all movement errors** | | | | | | | | | | | |
| --- | --- | --- | --- | --- | --- | --- | --- | --- | --- | --- | --- |
|  | **Abort pre-GO (% of initiated)** | | **Long RT  (% of GO trials)** | | **Long MVT  (% of GO trials)** | | | **Touch uncued  (% of GO trials)** | | **Touch distractor (% of GO trials)** | |
| **Monkey T** | 41.9 | | 1.3 | | 7.4 | | | 1.7 | | 17.4 | |
| **Monkey M** | 37.8 | | 4.4 | | 2.2 | | | 3.0 | | 22.3 | |
| **Number of correct behavioral trials for each color condition and movement direction** | | | | | | | | | | | |
|  | **Blue** | **Green** | | **Pink** | | **UR** | **LR** | | **LL** | | **UL** |
| **Monkey T** | 2261 | 1987 | | 1766 | | 1556 | 1647 | | 1577 | | 1234 |
| **Monkey M** | 2109 | 1821 | | 1643 | | 1396 | 1406 | | 1383 | | 1388 |
| **Proportion of distractor errors (% of correct + distractor)** | | | | | | | | | | | |
| **Monkey T** | 20.7 | 20.4 | | 16.7 | | 19.4 | 16.8 | | 18.6 | | 23.7 |
| **Monkey M** | 30.8 | 27.3 | | 11.2 | | 27.1 | 23.5 | | 23.9 | | 24.2 |
| **Reaction times in correct trials, from hand trajectories (ms)** | | | | | | | | | | | |
| **Monkey T** | 150 +/-40 | 152 +/-39 | | 147 +/-50 | | 150 +/-50 | 150 +/-38 | | 147 +/ 37 | | 152 +/-46 |
| **Monkey M** | 173 +/-57 | 169 +/-58 | | 153 +/-65 | | 170 +/-57 | 150 +/-62 | | 163 +/-60 | | 179 +/-59 |
