# Supplementary material for: Low and high beta rhythms have different motor cortical sources and distinct roles in movement control and spatiotemporal attention
Source: PLoS Biol. 2024 Jun 25;22(6):e3002670. doi: 10.1371/journal.pbio.3002670 (PMC11198906; doi:10.1371/journal.pbio.3002670)
Supplement: S2 Table — Related to Methods. Summary of recording reference positions for low and high beta dominant sites in each monkey, further separated for linear array probe and single-tip electrode sites. Tube—stainless steel body of Plexon probe; chamber—on screw of the titanium recording chamber, or in contact with chamber saline; headpost—skull screw of headpost. (DOCX) [file pbio.3002670.s012.docx]

|  | **Monkey T  (110 sites; 47 w. linear arrays)** | **Monkey M  (60 sites; 50 w. linear arrays)** |
| --- | --- | --- |
| **Low beta dominant sites** | Linear array: 20 tube; 10 chamber; 4 headpost  Single-tip: 30 chamber | Linear array: 34 tube; 2 chamber  Single-tip: 2 chamber |
| **High beta dominant sites** | Linear array: 8 tube; 3 chamber; 2 headpost  Single-tip: 33 chamber | Linear array: 14 tube  Single-tip: 8 chamber |
