# Supplementary material for: Low and high beta rhythms have different motor cortical sources and distinct roles in movement control and spatiotemporal attention
Source: PLoS Biol. 2024 Jun 25;22(6):e3002670. doi: 10.1371/journal.pbio.3002670 (PMC11198906; doi:10.1371/journal.pbio.3002670)
Supplement: Attachment — Submitted filename: NougaretKilavik_Rebuttal_R1_March2024.docx [file pbio.3002670.s016.docx]

**General comment:**

*Following the feedback and comments of the reviewers, we have slightly modified the structure of the manuscript.*

*The result section is now split into subsections. Each subsection has a small introduction and conclusion paragraph, helping the reading process. The figures corresponding to each subsection were reorganized accordingly, and 2 supplementary figures made after reviewers' suggestions were added to the supplementary material.*

**Reviewer #1:** This manuscript is of potential interest to the field of motor control in primates (including humans). As introduced in the manuscript, there has been a long-standing interest in the mu/alpha/beta band; however, there is confusion about the roles of the low vs higher frequency. This manuscript suggests that there may be differences in the premotor vs the motor regions. In general, this finding may be of interest to multiple fields. However, there are some fundamental methods details and analyses that needs clarification. Without these details, it is hard to fully understand the significance.

- *We thank the Reviewer for the positive feedback and the important questions below notably related to the distinction between beta and mu, and harmonics.*

1.	As the authors are aware, low and high beta could be simply be harmonics (consequence of fourier transform of a signal). For example, a strong mu (low beta) could have a waveform shape that also result in a high beta band harmonic. In other words, the closer a waveform deviates form a pure sine wave, the more there are harmomics at multiples. This might be suggested by Figure 2 and Figure 3A. Examining this in detail and showing waveforms is key. What are the LFP waveforms in each area? For PMd, is there really no fundamental mu band and only beta?

- *We agree with the reviewer that this is a very important issue. In our first analysis steps, we determined that low and high beta have different cortical sources, and generally different power modulation profiles across task epochs (Figs. 2-3). In order to avoid quantifying amplitude of the ‘aperiodic’ signal in our empirically-determined frequency ranges of interest, and in order to avoid spectral ‘leakage’ of the dominant band into the non-dominant frequency range, we decided to continue our analysis at that point using only the dominant band in each site. This indirectly to some extent also resolved the issue of harmonics, as high beta was only quantified further in sites with higher power for high beta compared to low beta, which would be unusual if this would be a harmonic of low beta (see also Rodriguez-Larios and Haegens 2023;* [*https://doi.org/10.56296/aip00006*](https://doi.org/10.56296/aip00006)*). Further substantiating our confidence that high beta is mainly not a harmonic of low beta in our study is the very different behavioral correlates of each band. To further strengthen the claim of independent fundamental frequencies, we now did several control analyses, which we show either in figures in this reply, or add as supplementary figures to the manuscript.*
- *First, we analyzed in more detail the example session shown in Fig. 2. Fig. R1A below shows the trial-averaged power modulation (for the full LFP signal, including the aperiodic component), averaged across the low (13-19 Hz) and high (21-30 Hz) beta frequencies. Clearly the average power modulation of the high beta band is similar in sites 1 and 2. Furthermore, the average power modulation of low beta is similar in sites 2 and 3. However, the modulations of power in the low beta frequency range in site 1 rather resembles that of high beta in that site, suggesting it could reflect spectral ‘leakage’ from the high to the low beta ranges. Also, the modulations of power in the high beta frequency range in site 3 rather resembles that of low beta in that site, suggesting it could either reflect spectral ‘leakage’ or potentially a harmonic. Fig. R1B shows the full power spectra in the pre-SC1 epoch for these three sites. Clearly, there is no peak in the low (mu) beta range for the anterior high-beta dominant site (site 1). For the intermediate site (site 2), both low and high beta are present, and after flattening (removing the aperiodic component) peak for high beta exceeded that of low beta, so in the paper we only analyzed further high beta in this site. In site 3, low beta dominated, but the power spectrum had a shoulder (bump) for high beta. High beta peaked at about 26 Hz in sites 1 and 2, while low beta peaked at about 15 Hz in sites 2 and 3. We next plotted single-trial flattened (periodic-only) power spectra in each site (Fig. R1C), highlighting the individual trials with highest power in either low or high beta frequency ranges. Trials which had very large power in low beta (sites 2 and 3) did not systematically also have a 2nd bump for high beta. Finally, we quantified waveform shape for the three sites (Fig. R1D). Interestingly, the rather isolated high beta band in site 1 had somewhat asymmetric rise/decay and peak/trough distributions, suggesting slightly longer durations spent in period rise flank and in period peak. The distributions were much broader in sites 2 and 3, but rather symmetric for rise/decay and peak/trough. We take this as an indication that these sites did not have a mu waveform shape (with strong presence of harmonics). Thus, in this example session, we conclude that site 1 mainly contained an isolated high beta band, and site 2 contained fundamental (independent) low and high beta bands. In site 3, we cannot completely rule out that high beta partly was a harmonic of low beta, but we did not analyze further high beta in sites with low beta dominance.*
- *Second, to examine directly the possible relation between high and low beta we exploited the sessions in which a high dominant site was recorded simultaneously with a low dominant site (n=26 in Monkey T / n=9 in Monkey M). We performed a cross correlation analysis as in Figure 7 C-E, i.e. the equivalent of a joint peristimulus time histogram applied to high and low beta amplitude along the trial. We did not observe any consistent correlation between the two bands. This control analysis was now added to the manuscript as Supplementary Figure S5.*

A. Trial-averaged beta power for low and high beta frequency ranges

B. Full power spectral density in pre-SC1 period

C. Periodic-only power spectral density in single trials in pre-SC1 period

D. Beta waveform cycle features

**Figure R1**. Detailed beta band characterization in the example session shown in Fig. 2 of the manuscript. A) Temporal profile of trial-averaged power, averaged for each beta frequency range (Low Beta: 13-19 Hz and High Beta: 21-30 Hz) and normalized in each site by the mean power in the full beta range (13-30 Hz). B) Trial-averaged power spectral density (PSD) in the pre-SC1 epoch. Single trial PSDs were estimated with Welch’s method, then averaged across trials for each site. C) Single-trial PSD calculated in the same epoch as B, after removing the aperiodic part of the signal. All trials of each site are plotted in gray, and the 30 trials with highest peak in the power (~18% of total trials per site) are color-coded, the color corresponding to either a stronger peak in the low beta range (orange) or high beta range (blue). In A-C, the three subplots correspond to sites 1, 2, and 3 (from left to right).

D) Histograms of waveform features obtained with the by-cycle toolbox (Cole SR & Voytek B, 2019; <https://doi.org/10.1152/jn.00273.2019>) from sites 1, 2 and 3. Cycles between 13-30 Hz were analyzed after initial low-pass filtering at 120Hz (4 times the maximum frequency of interest). We defined the following thresholds: 'amp_fraction_threshold': 0.6, 'amp_consistency_threshold': .3, 'period_consistency_threshold': .5, 'monotonicity_threshold': .6 and 'min_n_cycles': 3, based both on visual inspection and values suggested for motor cortex recordings in Cole SR & Voytek B, 2019. In all histograms, only cycles that were considered bursts have been plotted. Left: distributions of cycle duration for each site computed across all trials. Middle: distributions of rise-decay asymmetry, measured as a fraction of the period corresponding to the rise time. A value of 0.5 would indicate the same rise and decay time (vertical dashed line). Right: distributions of peak-trough asymmetry, i.e. the time fraction corresponding to the peak from peak and trough time. Equal duration of peak and trough would correspond to 0.5 value (vertical dashed line).

2.	The beta band index is particularly sensitive to point #1. How does waveform shape relate to this index.

- Indeed, following the aspects explored in relation to point 1, waveform shape, and in particular the presence of harmonics, could affect the beta index. In particular, in sites with the coexistence of both low and high beta as fundamental frequencies, any harmonic of the low beta falling into the high beta frequency range could add to the power of the fundamental high beta band, to artifactually bias the index in favor of the high beta band.
- In the example sites explored above with either comparable low and high power (site 2), or dominant low beta (site 3), the waveform rise/decay distributions (or peak/though distributions) were not particularly skewed, but rather symmetric, suggesting only weak, or at least not any systematic presence of harmonics resulting in a stereotypical mu rhythm waveform.
- In general, the high beta band peak frequency was not a multiple integer of the low beta peak frequency. Furthermore, even in sites with comparable low and high beta power (e.g. Fig. 2 example site 2, also explored above) the two bands modulated differently with the task. This was furthermore mirrored in the very different average MUA modulations in low and high beta dominant sites, suggesting sites were mainly well classified. Thus, even if the exact values of the beta band index in some intermediate sites could be spuriously biased in favor of high beta, the overall claims of the paper should remain robust.

3.	I was confused by the methods and whether there was referencing or not. Given all the types of electrodes used, this is important. Please make a table with each animal/recording approach/note of GND and referencing or not. This can have important effects on the LFP signal ◊ then will affect filtering.

- *We agree with the Reviewer that referencing is an important issue that might affect the signal. To clarify this point, as suggested by the Reviewer, we have now made a table (new Table S2 in the Supplementary Materials) where we summarize the different referencing points used across electrode types, for each monkey. We furthermore added a supplementary sentence on page 26 to clarify further the referencing for the LMA (Alpha Omega linear arrays).*
- *During data analysis, we did not detect any obvious differences (in overall LFP signal, nor beta band dominance) between sites recorded at adjacent/comparable cortical locations but with different references, e.g. using a very local reference (Plexon probes with reference on the individual probe stainless steel body entering the local cortical column) or using a common joint reference/ground on the (still local) titanium recording chamber for sessions with multiple single-tip electrode sites. Notably, low and high beta sites were observed with each of the referencing schemes, and in both single-tip electrodes and linear arrays (Table S2).*
- *Note that since the Plexon probes with stainless steel body were inserted through the dura, and since in the titanium recording chamber we always applied saline to improve signal quality and preserve the humidity of the dura, we essentially also referenced to the saline and recording chamber for these probes.*
- *The only sites that differed substantially in reference location are the handful for which we referenced to the distant headpost. This subset is too small to make any substantial change to the conclusions made in this study.*
- *Please see also the reply to a related comment from Reviewer #2.*
- *Preparing this table allowed us to detect and rectify minor numerical typos in the text regarding the referencing, on page 26.*

4.	Using phase for local effects might also be problematic because of the harmonic and the phase consistency b/w high and low beta. The authors should examine cross-frequency (bi-spectra) between these two bands per channel. Is there a consistent phase b/w the two?

- *This point is also related to one point raised by Reviewer #2. For phase-locking analysis it is important to first verify that the frequency of interest contains a spectral ‘peak’, and indeed also that this is not an harmonic of a lower band. As we explained to Reviewer 2, for this reason we only analyzed locking to the locally dominant band. Thus, in sites where we quantified locking to the high beta band, this band had higher power than the low beta band. This, and furthermore the fact that low and high beta amplitude modulated very differently with the task and the behavior, makes it unlikely that harmonics had any strong effect on phase locking quantification in our study.*

5.	Was there any food or water restriction or positive rewards? This was not clear from the methods.

- *We previously mentioned in the methods that we used positive reinforcement (liquid reward; page 25). We now have also added that they were on a restricted and controlled water regime (including fruit and vegetables), with free access to dry food (page 22).*

6.	Can the authors show example plots of the movement threshold. The values in Figure 1 imply velocities of 0.2 and 0.6 cm/s.

- *On page 29 we detailed the threshold used for determining the center-out reaching movement onset (“... in a 2000ms duration epoch centered on GO, periods with prolonged increased velocity (>50ms) above an empirically determined velocity-threshold (6 cm/s) were then detected, and the final, preceding increase in acceleration above an empirically determined acceleration-threshold (6 cm/s/s) was then taken as the time of movement onset.”).*
- *In the single trial examples in Suppl. Fig. S1B, the thus detected movement onset was indicated with a black dot for each trial.*

7.	For some reason, I could not find figure legends. Thus, not clear what all the elements of the figure mean. Might be easiest if they are part each figure.

- *We are really sorry for the inconvenience caused by the lacking legends. By a mishap during manuscript submission, we removed the legends when we removed the figures at the end of the manuscript, as we decided to instead upload the figures independently as high quality pdf files in the submission process. The legends were subsequently submitted after the request from one of the reviewers via the editorial office. The legends are now attached in the revised manuscript.*

**Reviewer #2**: This is a very nice paper, highlighting the distinction between low and high beta more clearly than any other paper I know. I have mainly interest-driven comments - the paper actually looks pretty good to me as it is.

- *We thank the Reviewer for the positive feedback and the interesting comments below notably regarding additional aspects to explore in the data.*

Comments:

I assume that the animals' choice could be equally well predicted from pre-GO velocity, i.e. the onset of micro-movements? This would emphasize the influence of motor processes on choice decoding, which I feel is sometimes underrated.

- *This comment is in line with several recent papers that highlight the abundance of task-correlated ‘fidgeting’ movements in human and animal behavior during task performance, and the strong reflections of these uninstructed movements in neuronal activity across most of the brain. It could even potentially be that such motor processes have a causal influence on choice decoding as the reviewer seems to suggest, but at the very least, it seems to strongly correlate with it.*
- *In our study, the capacity to decode color conditions using the amplitude of either low or high beta (Fig. 4-5) should probably not be interpreted as if the motor cortex contains representations of each color condition per see. Rather, the correlations that we observe between each beta band and distinct uninstructed behavioral parameters such as gaze position and hand micro-movements, which themselves are modulated by the color conditions (i.e. by the different temporal structure of task-relevant information in each condition) might be strong drivers of the beta decoding capacity.*
- *We now directly tested decoding the color condition using spontaneous hand and eye movements. Indeed, the decoding is robust with any of the two signals. As these results were so clear, and added to the interpretation of possible causes for the color condition information carried by low and high beta, we now added this spontaneous behavior decoding as a supplementary figure (Fig. S2) which we also refer to in the results (page 7) and in the discussion (pages 16, 19 and 21).*

I wonder if the authors found any gamma responses/oscillations and, if so, whether these gamma oscillations behaved like a negative image of high-beta.

- *We did not observe any clear narrowband gamma in this dataset (see power spectra in Fig. S10, up to ~200Hz). However, broadband gamma (see Fig. R2 below), which might predominantly reflect population spiking activity, modulated in a similar manner as the MUA already shown in Fig. S6, hence at the grand trial- and site-averaged level quasi inverse to the locally dominant beta band. There were phasic increases in normalized broadband gamma power during each valid SC in the averages of the high beta dominant sites, and prolonged increased broadband gamma power between valid SC and GO, and further increased power during movement execution in the averages of the low beta dominant sites. Please note that as we mentioned in the manuscript, such an inverse average relationship between LFP beta amplitude and MUA (or broadband gamma) does not mean there’s necessarily an intrinsic, moment-by-moment interaction between these signals, nor between beta amplitude and neuronal firing rate at the level of single neurons (Confais et al. 2020).*

***Figure R2****. Normalized power in gamma and high-gamma frequency ranges (50-200Hz), in high (left) or low (right) beta dominant sites, split for the three color conditions (top->bottom). Power in each frequency was normalized to the average power across all trial-epochs in each individual site, before averaging across sites. Warmer colors indicate increased power.*

In Fig. 5, left column (high beta), some of largest differences between conditions arise before the SEL cue. At that time, the animal had no information about the kind of trial, right? So why do the brain oscillations distinguish between conditions at this stage?

- *Indeed, the high beta band distinguished between the three color conditions from very early in the trial, contrary to the low beta band.*
- *The monkeys were working with a block design, with small blocks of about 15 correct trials in each color condition before switching to a new color condition (described in page 24 in the Methods).*

It would be nice to see a grand-average power spectrum across high beta dominant and low beta dominant sites to appreciate how strong the separation really is.

- *We now added grand average spectrograms as subplot D to Fig. 3, after splitting all sites into 3 groups with either strong high or low beta dominance, or comparable strength of the two bands (within +/-0.25). We only show the strongly high and low dominant sites.*

In Fig S5, top row, the authors display a "full" model, including all selected predictors, to better understand the hierarchy of predictors. I assume it might be helpful to also see the influence of each predictor (coefficients) vs. time, in addition to the variance explained.

- *Indeed, in the original control analysis now in Suppl. Fig. S7, we scrambled one regressor at a time in the full model to see how much the explained variance dropped by effectively removing the information carried by that regressor.*
- *To answer the question of the reviewer more precisely, we now performed a complementary analysis in which the beta coefficient of each regressor vs time was computed in each of the 3 colors conditions. Note however that the coefficients associated to each regressor are influenced by the ratio between the number of observations for categorical variables (in our case the gaze position) and also by the variance of continuous variables (the hand velocity had a much lower variance from between SEL and GO, and a larger variance at the beginning and the end of the trial).*
- *We exemplified these potential biases in considering the coefficients in the figure below (Fig. R3). The modulation of low beta by the hand velocity is illustrated. In the ‘’full’’ linear model and as in Fig. S7, four regressors were considered (Reaction time, Hand velocity, Gaze position and Time on task). For each temporal bin (n=680), we extracted 3 parameters informing on the modulation of the low beta amplitude by the hand velocity: the coefficient, the percentage of variance explained and the p-value. The coefficients were not directly correlated with the percentages of variance explained (Fig. R3, top left) nor with the p-values (Fig. R3, top middle). However, the percentages of variance explained and the p-values were directly correlated (monotonous relationship): the lower the p-values, the higher the percentages of variance explained (Fig. R3, top right). Consequently, we considered that the percentage of variance explained was a more informative measure than the coefficient. At the bottom of the figure, the beta coefficient (left), the percentage of variance explained (middle) and the p-values (right) are plotted along the task events.*

***Figure R3****: Low beta modulation by hand velocity in the blue trials. A linear model analysis was performed and the beta coefficients, the percentages of variance explained and the p-values were computed. The 3 subplots on the top show the 2-by-2 relations between these 3 parameters. Only for the last correlation there was a consistent (monotonic) relationship between the two parameters: a higher percentage of variance explained meant a lower p-value. The 3 plots at the bottom show the behavior of these 3 parameters along the task. Vertical and horizontal red lines show the significance level used in the manuscript (0.01).*

Minor:

"For the high band dominant sites, 47.7% of neurons (27/60 neurons in monkey T and 45/91 in monkey M) were significantly phase-locked to high beta phase. For the low band dominant sites, 12.3% of neurons (22/269 neurons in monkey T and 38/218 in monkey M) were significantly phase-locked to low beta phase." The number of units locked to the respective "non-dominant" rhythm should also be reported.

- *This question is somewhat related to the last comment of Reviewer #3, and also to point 4 of Reviewer #1.*
- *First, when considering this question, we discovered two small typing errors in the manuscript that we corrected (page 8).*
- *All through the analysis for this paper, we considered carefully how to treat the non-dominant beta band. There were sites with clearly only one peak in the power spectrum for either high or low beta (as the example sites in Fig. 2A and C), or sites with two bands but with task epochs in which only one band was present (as the example site in Fig. 2B, in epochs after the onset of the valid cue when low beta power drops). In both cases, trying to quantify the amplitude or phase of a frequency range in the absence of any clear peak in the power spectrum means we are in reality analyzing the aperiodic signal within that frequency range, which does not readily make sense.*
- *Furthermore, such amplitude or phase estimates might be ‘contaminated’ by the presence of the other, dominant beta band with potentially much stronger power, since the low and high beta bands were only separated by 10 Hz on average.*
- *Thus, any phase locking analysis to the non-dominant band might only be attempted in the sites with relatively weak dominance (e.g. such as the example site in Fig. 2B), and in a trial epoch with clear presence of both bands (pre-SC1 as we already used).*
- *We tested this for the linear array sites with weak beta dominance indices within +/- 0.25. Given the presence of both bands in these sites, as expected we also found neurons locked to the non-dominant band. Collapsed for the two monkeys for these weakly dominant sites, this corresponds to 12.4% locked to low in low-dominant sites, 18.0% locked to low in high-dominant sites. 52.5% locked to high in high-dominant sites, and 13.5% locked to high in low-dominant sites.*
- *However, these percentages represent limited numbers of neurons (Monkey T: 148 units in low beta dominant sites, of which 15 locked to low and 15 to high; 35 units in high beta dominant sites, of which 19 locked to high and 7 locked to low beta. Monkey M: 45 units in low beta dominant sites, of which 9 locked to low and 11 to high; 26 units in high beta dominant sites, of which 13 locked to high and 4 locked to low beta). We therefore prefer to not include these numbers, which only account for a subset of sites, in the manuscript.*

It seems that the referencing was quite variable across recordings. Why was it changed so often?

- *This question was also raised by Reviewer 1. Please see also our response to this Reviewer. We have now made a small table (Supplementary Table S2) to summarize the different references used.*
- *First, single-tip electrode referencing was different from that of laminar probe recordings, as we mainly used the standard methods for each type of electrode/probe. Furthermore, the AlphaOmega LMA laminar probe was different from the Plexon laminar probes since it did not have the option of ‘internal’ referencing to the probe's stainless steel body, as its probe body was mainly epoxy. For the LMA we therefore used a method comparable to the single-tip electrode recordings. In all these cases, the referencing remained rather local, either common on the recording chamber or in the liquid within the chamber above the exposed dura, or very local within the cortical column.*
- *The few sites obtained with distant referencing (6 Plexon V-probe sites in monkey T, for which low and high beta dominance were observed), were originally done to explore to which degree this made substantial changes to the recorded signal. As we did not note any appreciable difference for the beta, compared to local referencing, we decided to include these sites in this study.*

p. 32: "Next, the power matrix (trial x window x frequency) for each LFP was normalized by dividing by the mean power between 10-40Hz across trials and windows for that LFP."

Was the same value used to normalize power at all frequencies of the time-frequency spectrum? Usually, one would prefer a normalization per frequency.

- *This normalization was only used for visualization in power spectra and spectrograms (Figs. 2 and 3), not for any data analysis. In each site, we normalized the whole spectrogram by 1 value, calculated across the whole frequency range of interest.*
- *We normalized in this way to allow 1) preserving the respective amplitude differences between the low and high beta, and 2) still be able to combine sessions which could be very different in overall signal amplitude.*
- *For the beta amplitude analysis (notably correlations with task and behavioral factors, combining sites and monkeys), we calculated in single sites the Hilbert amplitude after filtering for the dominant beta band. For each site, the trial-cut amplitude was then normalized (subtracting mean and dividing by standard deviation), before lumping all sites (as described on manuscript page 37), to have comparable overall amplitude across sites, so as to focus on the modulations in amplitude across conditions and trial epochs.*
- *We now also noticed that we originally described the Methods for decoding task condition with beta amplitude (which used these normalized amplitudes) before we described the normalization in the section below (LM and cross-correlation analysis). We corrected this by moving the “Preprocessing for beta amplitude analysis” subsection before the “decoding procedure” subsection in the Methods.*

p. 25, bracket missing: "Continuous hand position (X and Y) was obtained from two perpendicularly superimposed contactless linear position magnetorestrictive transducers,model MK4 A; GEFRAN, Provaglio d'Iseo, Italy)."

- *Thanks!*

**Reviewer #3**: Review for 'Distinct sources and behavioural correlates of macaque motor cortical low and high beta' by Nougaret and colleagues for PLOS Biology

Summary

The authors investigate beta activity over 59 and 39 sessions using multi-electrode recordings from two adult male rhesus monkeys performing a complex visuomotor task.

The authors report distinct beta activity:

1.	low beta located in M1 which correlates positively with reaction time and negatively with micro-movements

2.	high beta located in the dorsal premotor cortex which relates to task prediction.

First, I want to apologize to the authors and to the editor for the slight delay - I needed to sleep on this one a bit. I also would like to point out that I'm not an expert in the acquisition and analysis of eye position data.

I think this is excellent work in an interesting line of questioning. The paper is generally well-written, and the visual illustrations are exceptional.

- *We thank the Reviewer for the positive feedback and the comments below on how to improve readability and interpretation of the results.*

However, despite reading the results section several times, I find it quite hard to grasp all the information and make sense of why specific analyses were conducted. One aspect that contributed to this is that the results are presented prior to the methods. I understand that this is journal-dependent rather than the author's choice. However, I would highly recommend adding enough information in a short and compact way to the respective results section. This will increase the flow and prevent the reader must jumping back and forth between the results and methods sections.

- *There were many points we felt essential to include, we therefore appreciate the suggestion from the reviewer on how to increase the readability of the results!*
- *We have now added short summaries for analysis selection and the conclusions of each analysis at the start and end of several results sections.*

Further, I wonder if some results could be moved to the supplementary material.

- *We considered carefully each analysis and results which were described as either main or supplementary figures. We however think we found the optimal compromise between the main results that we feel are needed to make the complete story, and the supplementary materials that substantiate these findings with additional results or complementary methods.*

Finally, I strongly feel that each analysis should be well-motivated and summarised. Adding systematically two sentences: one sentence before an analysis explaining why the following analysis was conducted; and one sentence after an analysis summarising what this analysis yield would be extremely helpful. At times the results section reads like a collection of analyses that are presented back-to-back, whereby the reader is sometimes wondering why a particular analysis has been conducted.

- *Again, we thank the reviewer for this comment. As mentioned above, we have now made an effort to improve the readability by motivating and summarizing better each step of the analysis.*
- *Notably, we took the occasion to restructure the whole results section, and some of the figures, to better group related analyses, and make natural distinctions between each set of analyses.*
- *In particular, we reconfigured the figures illustrating the decoding and linear models results (originally 4 and 5; 4 to 6 in the revised version) and modified the text in several spots in the Results section.*

I am a bit perplexed by the very different time courses of low and high beta (for instance as presented in Fig. 4A). If I understood correctly the authors present data from different frequencies (low, high) at different locations (M1, PMd). However, there is no figure showing the low beta in PMd and the high beta in M1. I think it would be useful to show these data, as well as statistically compare the time courses of low and high beta in the same spatial location. This allows unpicking if the reported differences between low and high beta arise from different spectral properties of the signal, the different spatial properties of the signal, or both. To me, this is a very important point the paper should incorporate given the title and claims of the paper.

- *We thank the reviewer for this comment. This also relates to a comment from reviewer 1 regarding harmonics, and reviewer 2 regarding phase-locking of spikes to the non-dominant beta band. Please see also our replies to these points above.*
- *The beta band dominance index changed gradually across the cortical surface (Fig. 3C), but with clear dominance of high beta in a majority of anterior sites and low beta in a majority of posterior sites. At intermediate sites both bands had comparable amplitudes. Following a request of Reviewer 2, we now added grand average spectrograms to Figure 3 (Fig. 3D), after splitting all sites into 3 groups with either strong high or low beta dominance, or comparable strength of the two bands (indices within +/-0.25). We only show the strongly high and low beta dominant sites in these new plots (see also example sites in Fig. 2A-C). Clearly in the sites with strong dominance of one band, the other band was largely absent. Thus, our general claim that there is a double-dissociation between cortical source and behavioral correlates of these two bands seems warranted.*
- *Note however that we did not attempt to formally define a border between M1 and PMd in this study (which could maybe have been attempted based on thresholds for inducing movements with ICMS, or based on reconstruction of sulcal landmarks underlying the estimated recording chamber location using MRI). The definition of the M1/PMd border varies across studies in the literature, mainly due to the fact that it is rather gradual, rather than abrupt in the sense of anatomical or neuronal response properties. We apply the labels M1 and PMd rather loosely to define more anterior and posterior recording sites, respectively, while for intermediate sites, any clear cortical area assignment is rather fussy. Interestingly, the gradual change in the beta band index parallels this gradual anatomical and functional transition from M1 to PMd.*
- *As we already discussed in reply to reviewer 2, regarding the analysis of phase locking to the non-dominant band, the quantification of the non-dominant band is not straightforward for theoretical and practical reasons. Trying to quantify the amplitude of a frequency range in the absence of any clear peak in the power spectrum means we are in reality analyzing the aperiodic signal within that frequency range, which does not readily make sense. Furthermore, such amplitude estimates might be ‘contaminated’ by the presence of the other, dominant beta band with potentially much stronger power, since they were only 10 Hz apart on average (spectral leakage). Finally, regarding sites with low beta dominance, any high beta component could potentially reflect a harmonic, as justly pointed out by Reviewer 1.*

Check the spelling of occured > occurred

- *Thanks!*
